# Antisense suppression of donor splice site mutations in the dystrophin gene transcript

**DOI:** 10.1002/mgg3.19

**Published:** 2013-06-13

**Authors:** Sue Fletcher, Penny L Meloni, Russell D Johnsen, Brenda L Wong, Francesco Muntoni, Stephen D Wilton

**Affiliations:** 1 Centre for Comparative Genomics, Murdoch UniversitySouth St, 6150, Perth, Western Australia, Australia; 2 Centre for Neuromuscular and Neurological Disorders, University of Western Australia Perth6009, Western Australia, Australia; 3 Department of Pediatrics, Cincinnati Children's Hospital Medical Centre and University of Cincinnati College of MedicineCincinnati, 45229-3039, Ohio; 4 The Dubowitz Neuromuscular Centre, University College London Institute of Child Health LondonLondon, WC1N 1EH, United Kingdom

**Keywords:** Duchenne muscular dystrophy, exon skipping, splice mutation, splice-switching oligomer

## Abstract

We describe two donor splice site mutations, affecting dystrophin exons 16 and 45 that led to Duchenne muscular dystrophy (DMD), through catastrophic inactivation of the mRNA. These gene lesions unexpectedly resulted in the retention of the downstream introns, thereby increasing the length of the dystrophin mRNA by 20.2 and 36 kb, respectively. Splice-switching antisense oligomers targeted to exon 16 excised this in-frame exon and the following intron from the patient dystrophin transcript very efficiently in vitro, thereby restoring the reading frame and allowing synthesis of near-normal levels of a putatively functional dystrophin isoform. In contrast, targeting splice-switching oligomers to exon 45 in patient cells promoted only modest levels of an out-of-frame dystrophin transcript after transfection at high oligomer concentrations, whereas dual targeting of exons 44 and 45 or 45 and 46 resulted in more efficient exon skipping, with concomitant removal of intron 45. The splice site mutations reported here appear highly amenable to antisense oligomer intervention. We suggest that other splice site mutations may need to be evaluated for oligomer interventions on a case-by-case basis.

## Introduction

Duchenne muscular dystrophy (DMD), a fatal X-linked muscle wasting disorder of childhood, is caused by mutations in the dystrophin gene (*DMD)*, most commonly frame-shifting deletions of one or more exons (for review Emery (2002)). The milder allelic condition, Becker muscular dystrophy (BMD) is also caused by dystrophin mutations, but these are typically in-frame deletions that allow synthesis of internally truncated dystrophin isoforms that retain partial function. Characterization of dystrophin gene arrangements in mildly affected and asymptomatic BMD individuals has revealed that deletion of substantial domains of the *DMD* gene can yield dystrophin isoforms of near-normal function. These observations led to the concept of targeted exon removal around a DMD mutation to reframe the dystrophin transcript, and the supposition that such a strategy could treat DMD.

Splice-switching antisense oligomers were first used by Dominski and Kole (1993) to modify splicing of the β-globin transcript, and subsequently shown to induce dystrophin exon 19 skipping and reading frame disruption (Takeshima et al. 1995). Proof of concept that targeted exon removal could restore dystrophin expression in vivo was demonstrated in *mdx* mice (Mann et al. 2001), a dog model of DMD (Yokota et al. 2009) and more recently in DMD patients (van Deutekom et al. 2007; Kinali et al. 2009; Cirak et al. 2011; Goemans et al. 2011). Oligomer design and clinical studies have focused on removing exons that flank frame-shift deletions in the two dystrophin deletion “hotspots,” however, numerous motifs control exon selection and splicing, and mutations in any of these can ablate gene expression and cause disease. Splice motif disruption prevents proper exon selection or results in the use of cryptic splice sites that cause partial exon loss or intron retention, and may generate multiple aberrant transcripts (Fernandez-Cadenas et al. 2003), while deep intronic mutations can activate pseudoexon inclusion in the mature gene transcript. Although pseudoexon activation in the dystrophin gene transcript is a rare event, this is the only type of gene lesion for which targeted exon skipping would lead to a normal full-length gene transcript. Gurvich and colleagues reported two DMD cases caused by the inclusion of pseudoexons, derived from introns 11 and 45, and subsequent oligomer-induced skipping of the aberrant exons (Gurvich et al. 2008).

Dystrophin splice site mutations have been relatively neglected as targets in DMD therapeutic exon skipping studies, despite reports that splice motif changes cause at least 15% of all mutations in human inherited disease (Krawczak et al. 1992) and in an early study, 7% of DMD/BMD cases (Roberts et al. 1994). Correct exon selection requires basic cis-acting elements important in exon recognition, and canonical splice sites, embedded within the context of noncanonical sequence that is conducive to splice site recognition and binding of splicing factors (Krawczak et al. 2007). Mutations to both canonical and noncanonical sequences can weaken splice site recognition and may result in exon skipping, but the consequences are difficult to predict (De Conti et al. 2012). Donor splice site definition is a key step in splice site recognition and mutations affecting the exon–intron junction are reported to result in exon skipping when the immediate vicinity is devoid of alternative splice sites (Krawczak et al. 2007).

We report the use of splice-switching antisense oligomers (AO) to by-pass two donor splice site mutations, one involving exon 16 (DMD-16ss) and the other impacting upon exon 45 processing (DMD-45ss). The inactivation of these donor splice sites did not lead to exon skipping, as may be expected (Krawczak et al. 2007) but instead caused retention of the downstream introns. Despite this similarity, one mutation responded to single exon skipping while the other required dual exon skipping to overcome the disease-causing mutation and restore the open reading frame. As we have found with other small dystrophin gene lesions, it appears that the various dystrophin splice site mutations will require personalized oligomer design and exon skipping strategies on a case-by-case basis (Forrest et al. 2010; Fragall et al. 2011; Adkin et al. 2012).

## Materials and Methods

### AO design and synthesis

2'-O-methyl (2OMe)-modified bases on a phosphorothioate backbone were synthesized on an Expedite 8909 synthesizer (Applied Biosystems, Melbourne, Australia), as described previously (Adams et al. 2007). AO nomenclature is based upon target exon number and oligomer annealing coordinates as described by Mann et al. (2002), and oligomers to the most amenable sites were prepared and supplied by Sarepta Therapeutics (Bothell, WA) as phosphorodiamidate morpholino oligomers conjugated to a cell penetrating peptide (PPMO-*k*) ((RXR)_4_XB, where B = β-alanine; R = l-arginine; X = 6-aminohexanoic acid) (Moulton et al. 2007, 2009; Jearawiriyapaisarn et al. 2008; Moulton and Moulton 2010). Detailed design and optimization of oligomer sequences targeting exon 16 for removal were reported previously (Harding et al. 2006) and the exon 44, 45, and 46 oligomer sequences are shown in Table 1.

**Table 1:** Splice-switching oligomer sequences targeting exons 16, 44, 45, and 46

Coordinates	Sequence (5'→3')	Patent number
H16A(−12 + 19)	CUAGAUCCGCUUUUAAAACCUGUUAAAACAA	WO 2011/057350 A1
H44A(+65 + 92)	UGAGAAACUGUUCAGCUUCUGUUAGCCA	WO 2011/057350 A1
H45A(−03 + 22)	GCCCAAUGCCAUCCUGGAGUUCCUG	WO 2011/057350 A1
H46A(+93 + 122)	GUUGCUGCUCUUUUCCAGGUUCAAGUGGGA	WO 2011/057350 A1

### Cell culture and myogenic conversion of fibroblasts

All normal and patient biopsy samples were collected with informed consent and were deidentified. The use of human tissue in this study has been approved by the University of Western Australia Human Ethics Committee (approval number RA/4/1/2295). Fibroblasts and myogenic cells from normal and DMD patient muscle biopsies were expanded and differentiated as described previously (Adkin et al. 2012). Cells from the patient DMD-16ss, carrying the exon 16 donor splice site were obtained from a muscle biopsy, while dermal fibroblasts were prepared from a skin biopsy from the DMD patient (DMD-45ss) with an exon 45 splice site mutation. Normal myogenic cells were extracted from surplus muscle biopsy fragments from patients undergoing elective procedures, with informed consent. Fibroblasts were converted to myoblasts through forced myogenesis (Lattanzi et al. 1998) by transduction with a MyoD expressing adenovirus, Ad5.f50.AdApt.MyoD (The Native Antigen Company, Oxford, U.K.), and then differentiated in low serum media. Briefly, patient fibroblasts were cultured until 80% confluent, washed with phosphate buffered saline (PBS), detached with 0.25% Trypsin (w/v) (Gibco, Life Technologies), inactivated with medium containing 10% FCS, pelleted by centrifugation at 600 *g* and then resuspended in DMEM (Life Technologies) supplemented with 5% horse serum. Ad5.f50.AdApt.MyoD was added at a multiplicity of infection of 200 and the cells were seeded at 30,000 cells per well in 24 well plates that had been sequentially pretreated for 1 h with 50 μg/mL poly d-lysine (Sigma, Melbourne, Australia) and 100 μg/mL Matrigel (BD Biosciences, North Ryde, Australia). Twenty-four hours later, the medium was replaced. The cells were allowed to differentiate for 96 h after forced myogenesis, before being transfected with oligomers. In our experience, forced myogenesis of fibroblasts is adequate for RNA studies, but is not always efficient enough to result in induced dystrophin levels that are detectable by western blotting, and is very dependent on the quality of the original cell preparation.

### Transfection of myogenic cells, RNA extraction, and nested RT-PCR

2OMe AOs were transfected as cationic lipoplexes with Lipofectamine 2000® (1:1 w/w) (Life Technologies, Melbourne, Australia) in Opti-MEM media (Gibco- Life Technologies) as per the manufacturer's instructions (Harding et al. 2006). PPMO-*k* was transfected into the cells, at concentrations indicated, and as described (Adkin et al. 2012). After incubation as specified, RNA was extracted and RT-PCR undertaken using ∼100 ng of total RNA as template for primary amplification using Superscript® III One-step RT-PCR system with Platinum Taq (Life Technologies) to amplify across specified dystrophin exons. After 30 cycles (myoblasts) and 35 cycles (MyoD converted fibroblasts) a 1 μL aliquot was removed and subjected to nested PCR for 30 cycles using AmpliTaq Gold (Applied Biosystems, Melbourne, Australia). Details of PCR primers (Geneworks, Adelaide, Australia) are shown in Table 2.

**Table 2:** PCR primer sequences and combinations used in this study

PCR primer	Sequence 5'→3'
Exon 1 Fo	CTTTCCCCCTACAGGACTCAG
Exon 7 Ro	CTTCAGGATCGAGTAGTTTCTC
Exon 1 Fi	GGGAGGCAATTACCTTCGGAG
Exon 7 Ri	CTGGCGATGTTGAATGCATGT
Exon 9 F	CGATTCAAGAGCTARGCCTAC
Exon 18 R	GGATCTCCAGAATCAGAAAC
Exon 12 F	GCGAGTAATCCAGCTGTGAAG
Exon 17 R	CCGTAGTTACTGTTTCCATTA
Exon 40 F	CTCTAGAAATTTCTCATCAG
Exon 51 Ri	GGTAAGTTCTGTCCAAGCCCGG
Exon 41 F	AGAGCAAATTTGCTCAGTTTCG
Exon 47 R	TTATCCACTGGAGATTTGTCTG
Exon 69 Fo	GCAAAAGGCCATAAAATGCAC
Exon 75 Ro	ACGGCAGTGGGGACAGGCCTTT
Exon 69 Fi	CCCATGGTGGAATATTGCAC
Exon 75 Ri	TGTTCGTGCTGCTGCTTTAGAC
Exon 13 F	CACGCAACTGCTGCTTTGGAAG
Exon 15 F	GATGCAGTGAACAAGATTCAC
Intron 16 R	TCTCTGAGATAGTCTGTAGCATG
Intron 16 F	GACTTTCGATGTTGAGATTACTTTCCC
Exon 21 R	GGCCACAAAGCTTGCATCCAG
Exon 20 R	CAGTTAAGTCTCTCACTTAGC
Intron 45 R 1	ATGCAAGAGCTTGGCAAAAGA
Intron 45 R 2	AAGCTTAAAAAGTCTGCTAAAATG
Intron 45 F	TTGTGTCCCAGTTTGCATTAAC
Exon 47 R	TTATCCACTGGAGATTTGTCTG
Exon 43 Fo	GCAACGCCTGTGGAAAGGGTG
Exon 45 Ro	CAGATTCAGGCTTCCCAATT
Exon 43 Fi	CAGGAAGCTCTCTCCCAGC
Exon 45 Ri	CCTGTAGAATACTGGCATCTGT

Outer primer combinations are shaded in gray, and inner primer combinations are indicated.

### Gel analysis, imaging, and sequencing

Amplicons were fractionated on 2% agarose gels in TAE buffer and relative exon skipping efficiency estimated by densitometry of the full-length and oligomer-induced PCR products on images captured by the Chemi-Smart 3000 system (Vilber Lourmat, Marne–la–Vallée, France) (Adams et al. 2007). The identities of induced transcripts were confirmed by direct DNA sequencing, undertaken by the Australian Genome Research Facility (Perth, Australia). Densitometric analysis used Bio1D software (Scientific Software Group, Provo, UT).

### Western analysis of dystrophin expression

DMD16-ss cells were transfected with oligomer sequences targeting the exon 16 acceptor splice site, prepared as PPMO-*k* (2 μmol/L) or a 2OMe AO cationic lipoplex (400 nmol/L), and incubated for 7 days before harvesting and western blot analysis (McClorey et al. 2006). Protein extracts (∼30 μg) from normal and DMD-16ss cells (oligomer treated and untreated) were loaded onto denaturing 3–10% gradient polyacrylamide gels. Electrophoresis and western blotting were performed using NCL-DYS2 (Novocastra Laboratories, Newcastle-upon-Tyne, U.K.) in a protocol derived from those of Cooper et al. (2003) and Nicholson et al. (1989), and was described in detail previously (Fletcher et al. 2006). Protein loading was standardized according to myosin heavy chain expression, assessed by densitometry on a Coomassie blue stained gradient gel (Fletcher et al. 2006). Images were captured on a Vilber Lourmat Chemi-Smart 3000 system using Chemi-Capt software for image acquisition and Bio1D software for image analysis.

## Results

**Figure 1:**
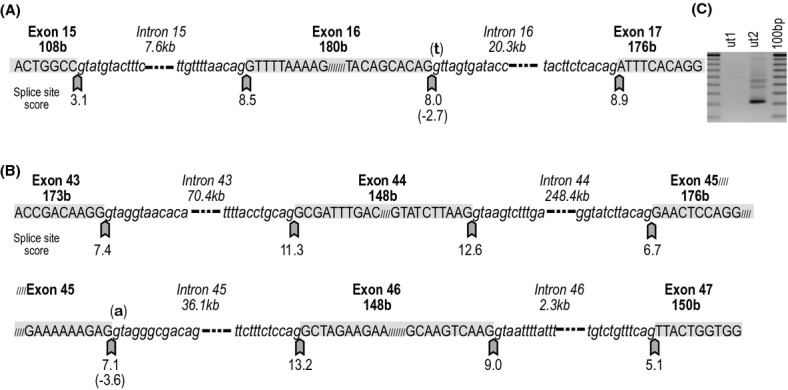
Arrangement of dystrophin exons (A) 15–17, and (B) 43–47 indicating exon (upper case):intron (lower case *italics*) sequences, intron length (shown in *italics*) and splice site scores, indicated by block arrows. Both mutations (c.1992 + 1 g>t and c.6614 + 1 g>a) occur at the invariant first “g” of introns 16 and 45, respectively. Splice site scores were determined using the algorithm http://rulai.cshl.edu/new_alt_exon_db2/HTML/score.html, and the scores resulting from the mutations are shown in parentheses. (**C**) RT-PCR across dystrophin exons 12–17 from RNA prepared from two independent untreated DMD-16ss myogenic cell cultures (ut). A 100 bp ladder was used as a size standard.

The exonic arrangement of the two regions of the dystrophin gene under investigation are shown in Figure 1, indicating exon:intron sequences, intron length, and splice site scores. Both mutations (c.1992 + 1 g>t and c.6614 + 1 g>a) occurred at the invariant first “g” of introns 16 and 45, respectively, ablating the canonical donor splice sites. Splice site scores were determined using the algorithm http://rulai.cshl.edu/new_alt_exon_db2/HTML/score.html that predicts the maximum 5' donor score as 12.6, with the average score of constitutive exons being 8.1. Hence, the normal exon 16 donor score of 8.0 is average (Fig. 1A), while the exon 16 donor splice site mutation lowered the value to −2.7. The normal exon 45 donor splice site (Fig. 1B) score of 7.1 was marginally below average, and the mutation reduced the value to −3.6. Dystrophin exon 16 is flanked by introns of 7.6 and 20.3 kb, whereas exon 45 is flanked by introns of 248.4 and 36.1 kb.

Cells from the two patients, DMD-16ss and DMD-45ss, were propagated and RNA extracted to assess the consequences of these mutations on the mature dystrophin gene transcript. RT-PCR across exons 12–17 (DMD-16ss) produced either no signal or sporadic amplicons of various lengths (Fig. 1C), while amplification across exons 41 to 47 (DMD-45ss) resulted in no product (data not shown).

### DMD-16ss studies

**Figure 2:**
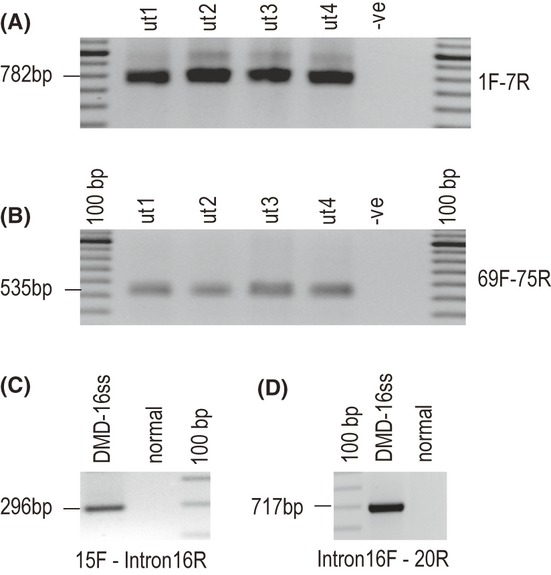
RT-PCR across dystrophin exons 1–7 (A), and 69–75 (B) on four separate RNA preparations from patient DMD-16ss. RT-PCR on RNA prepared from DMD-16ss cells using a forward primer directed to exon 15 and a reverse primer targeting intron 16 (C), and a forward primer annealing near the end of intron 16 and a reverse primer targeting exon 20 (D). RNA from normal human myogenic cells was included for comparison. A 100 bp ladder was used as a size standard.

The failure to generate consistent RT-PCR products across exons 12–17 from RNA extracted from untreated DMD-16ss cells, suggested that the dystrophin transcript was either absent, or present at extremely low levels. However, amplification across exons 1–7 and 69–75 confirmed that the dystrophin mRNA was indeed present, and could be readily detected under standard amplification conditions (Fig. 2A and B). RT-PCR was then carried out using primer combinations to ascertain if intron 16 was retained within the mature mRNA. A forward primer directed to exon 15 and a reverse primer targeting intron 16, as well as a forward primer annealing near the end of intron 16 and a reverse primer targeting exon 20 were used. RT-PCR products were generated from the patient RNA, corresponding to amplicons of the expected length if intron 16 was retained, and the remainder of the transcript had been correctly spliced (Fig. 2C and D). No such signals were generated from RNA extracted from normal human muscle and DNA sequencing of the exon 15F, intron16R product showed that dystrophin exon 15 was correctly spliced to exon 16, but the beginning of intron 16 was retained in the transcript (data not shown). Similarly, exons 17–20 were correctly spliced, but the end of intron 16 was included in the patient transcript, leading us to conclude that the 20.3 kb intron 16 was retained after splicing, expected to result in a full-length muscle specific dystrophin transcript estimated to be in excess of 34 kb (20.3 kb of intron retained within the 14 kb normal sized mRNA).

**Figure 3:**
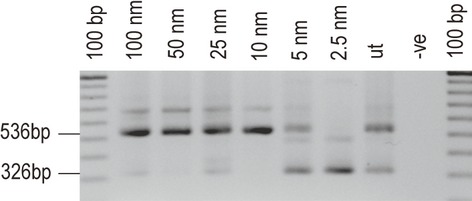
RT-PCR of RNA extracted from patient DMD-16ss cells transfected with a 2OMe modified antisense oligomer targeting exon 16, at concentrations indicated. The oligomer-induced amplicon missing exon 16 is 536 bp, and the smaller product (326 bp) is a revertant transcript missing exons 14–16 (reported previously Mitrpant et al. (2009)). The larger amplicon (∼700 bp) results from cryptic splicing, and is present in all samples.

After transfection of cells from patient DMD-16ss with a 2OMe-modified antisense oligomer targeting the exon 16 acceptor splice site, RNA was extracted and amplified by nested RT-PCR. As shown in Figure 3, dystrophin transcripts missing exon 16 were readily detected at all but the lowest transfection concentrations. An amplicon of near-normal length, detected in RNA extracted from untreated cells (Fig. 3, ut lane) was generated sporadically (Fig. 1C) and was previously identified as having arisen from activation of a cryptic exon 16 donor splice site (Mitrpant et al. 2009). The aberrant splice site is one base upstream of the correct splice site, and has a calculated donor splice site score of −4.7. The shorter amplicon, apparent in the untreated samples, and cells transfected at 2.5 and 5 nmol/L was previously reported as having arisen from spontaneous skipping of exons 14, 15, and 16 (Mitrpant et al. 2009).

**Figure 4:**
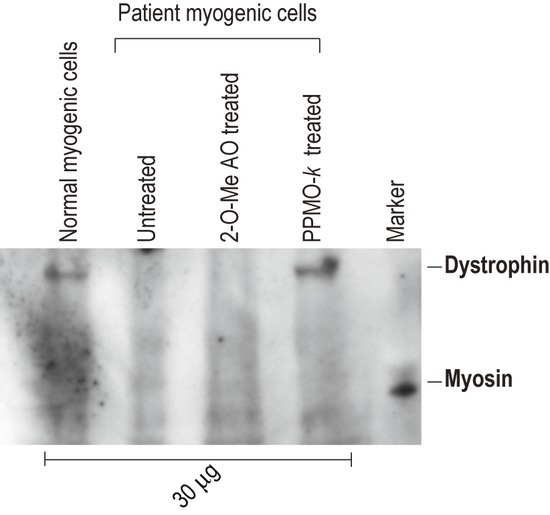
Protein extracts from normal cells and oligomer-treated and untreated DMD-16ss cells (∼30 μg, standardized to myosin) were analyzed by western blotting as described previously (McClorey et al. 2006). The 2OMe AO was transfected as a cationic lipoplex at 400 nmol/L and PPMO-*k* was added directly at 2 μmol/L, and the cells were incubated for 7 days. Dystrophin expression was revealed by NCL-DYS2 (Novocastra Laboratories) and chemiluminescent detection with Western Breeze (Life Technologies). Myosin (lower band) is indicated.

Dystrophin expression studies were undertaken on DMD-16ss myogenic cells, transfected with H16A(−12 + 19) prepared as 2OMe modified bases on a phosphorothioate backbone (2OMeAO), and as a phosphorodiamidate morpholino oligomer coupled to a cell penetrating peptide (PPMO-*k)*. Near-normal levels of dystrophin expression were detected in PPMO-*k* treated patient cells, whereas those treated with the 2OMeAO resulted in undetectable dystrophin expression under these conditions (Fig. 4**).** This is consistent with our previous experience, where dystrophin could not be induced to readily detectable levels in myogenic cells transfected with 2OMeAOs (Fletcher et al. 2006; McClorey et al. 2006). The absence of dystrophin in the untreated patient sample would indicate that the in-frame revertant fiber transcripts missing exons 14–16 were present at levels too low to generate detectable dystrophin.

### DMD-45ss studies

**Figure 5:**
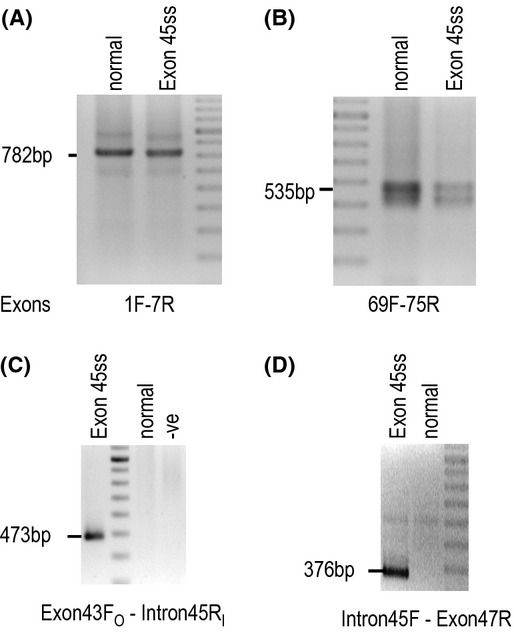
RT-PCR across exons 1–7 (A), and 69–75 (B) of the normal and DMD-45ss dystrophin transcripts. (C) RT-PCR using a forward primer targeting exon 43 and a reverse primer annealing within intron 45 and (D) a forward primer annealing at the end of intron 45 with a reverse primer targeting exon 47.

RT-PCR across regions of the DMD-45ss dystrophin transcript, remote from the exon 45 gene lesion, indicated that the mature dystrophin mRNA was present in the DMD-45ss cells (Fig. 5A and B). The failure to reproducibly generate RT-PCR amplicons across exons 41–47, suggested retention of intron 45, and this was confirmed by RT-PCR using a forward primer targeting exon 43 with a reverse primer annealing within intron 45, and a forward primer annealing at the end of intron 45 with a reverse primer targeting exon 47 (Fig. 5C and D). DNA sequencing confirmed the exon45:intron45:exon46 junctions (data not shown) and, assuming that intron 45 is retained in its entirety, would result in a 50 kb dystrophin transcript as a consequence of the exon 45 donor splice site mutation.

**Figure 6:**
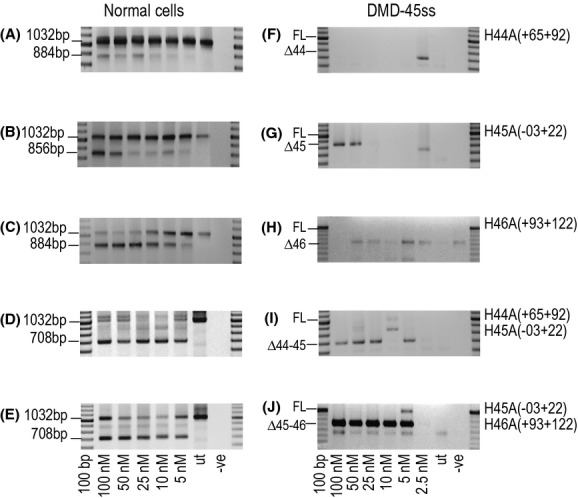
Normal (left panel) and DMD-45ss myogenic cells (right panel) were transfected with 2OMe phosphorothioate oligomers targeting single exons 44 (A, F), 45 (B, G) or 46 (C, H) or the dual exon blocks, 44 and 45 (D, I), or 45 and 46 (E, J), at concentrations indicated. Nested amplification used a forward primer in exon 41 and a reverse primer in exon 47 to generate a full-length transcript product of 1032 bases. The sizes of exon-skipped products are indicated. A 100 bp size standard was used to estimate amplicon sizes.

The excision of exon 45 alone disrupts the dystrophin reading frame, and hence it was necessary to excise either exons 44 and 45 or exons 45 and 46 from the mature mRNA to allow translation of a BMD-like dystrophin isoform. Single and dual oligomer preparations targeting these exons were transfected into normal myogenic cells, and dose-dependent induction of exon skipping is shown in Figure 6 A–E. Dual exon skipping of 44 and 45 and exons 45 and 46 appeared similarly efficient, with perhaps the cocktail targeting exons 44 and 45 being marginally more effective, as demonstrated by lower amounts of the full-length dystrophin transcript amplicon (Fig. 6D and E).

Oligomers targeting exons 44, 45, and 46: H44A (+65 + 97), H45A(−03 + 22) and H46A (+93 + 122), respectively, were transfected into DMD-45ss patient cells, individually and in combinations. Single exon excision did not generate detectable transcripts in the patient cells (Fig. 6F–H), apart from application of H45A(−03 + 22) at the transfection concentrations of 50 to 100 nmol/L, when amplicons of the expected size (missing exon 45) were observed (Fig. 6G). The inability to amplify products from DMD-45ss RNA appears to be due to the retention of intron 45 (36.1kb) in the patient dystrophin mRNA, resulting in a transcript target size that would preclude RT-PCR amplification.

Oligomer combinations targeting exons 44 and 45 and exons 45 and 46 were transfected into the DMD-45ss patient cells, and induced abundant transcript products missing the targeted exon combinations. Exon 45 and 46 exclusion was efficiently induced at all concentrations from 5 nmol/L and above, while exon 44 and 45 skipping appeared less effective, reflected by the amount of induced transcript and the presence of intermediate products in cells transfected with 2OMeAOs at 10 nmol/L or less (Fig. 6I and J).

**Figure 7:**
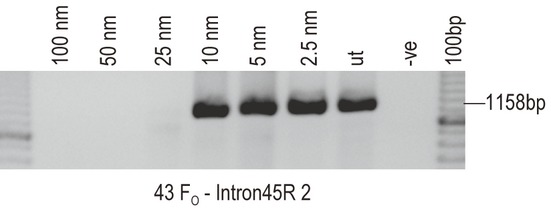
RT-PCR from exon 43 to intron 45 to detect dystrophin transcripts retaining intron 45, in the presence and absence of oligomers targeting exons 44 and 45. The size of the amplicon expected from retention of intron 45 is indicated (1159 bp).

RT-PCR to detect dystrophin transcripts retaining intron 45 confirmed that the failure to generate signals as a result of single exon skipping was due to the retention of intronic sequence. However, at transfection concentrations of 25 nmol/L and above, the skipping of exons 44 and 45 completely suppressed intron 45 retention (Fig. 7). Although transfection of patient cells with AOs targeting exons 45 and 46 induced robust exon skipping, intron 45 specific RT-PCR indicated the presence of transcripts retaining intron 45 at all concentrations (data not shown).

The forced myogenesis of dermal fibroblasts induces sufficient dystrophin expression to permit RNA studies, and, when cell quality permits, protein analysis by western blotting. However, in this case, the myogenic capacity of the MyoD transduced DMD-45ss patient cells was not adequate for dystrophin detection (data not shown).

## Discussion

Antisense oligomer-mediated exon skipping to reframe the dystrophin gene transcript is showing great promise as a potential therapy to overcome DMD-causing mutations. Dystrophin expression has been reported after systemic administration of splice-switching oligomers, of two different chemistries, in DMD patients (Cirak et al. 2011; Goemans et al. 2011). However, unlike gene or cell replacement approaches for which one treatment should be applicable to all DMD patients, regardless of the primary gene lesion, targeted exon skipping strategies must be tailored to the mutation, and may have to be personalized for some individuals. The first requirement is accurate genetic diagnosis, with precise identification of the gene lesion at the DNA, and preferably, the gene transcript level.

Mutation analysis at the DNA level can reliably identify gross genetic lesions and premature termination codons as causes of genetic disease, however, several other mechanisms can alter or disrupt gene expression, including alterations that affect the fidelity of pre-mRNA splicing (for review see Baralle et al. (2009)). The consequences of small insertions, deletions, or point mutations on the processing of the dystrophin gene transcript can be difficult to predict, and therefore, the clinical implications of such DNA changes are best determined by transcript analysis, although this is complicated by the necessity for RNA samples prepared from the appropriate tissues.

Disruption of canonical splice sites would be expected to disrupt splicing, however, the effects of mutations to noncanonical bases, branch points, and splicing regulatory elements are modulated by both *cis* and *trans* acting factors, and RNA secondary structure. An early estimate suggested that 15% of all human point mutations result in aberrant splicing (Krawczak et al. 1992), although this is now recognized as an underestimation for some genes (Baralle et al. 2009; Jensen et al. 2009). The importance of aberrant splicing as a cause of human disease has lead to advances in tools to predict the consequence of a particular nucleotide variation on gene expression (De Conti et al. 2012), but uncertainties in modeling such complex systems limits the accuracy of these predictions (Baralle et al. 2009).

The most common consequence of mutations affecting authentic splice sites is skipping of one or more exons, followed by aberrant donor or acceptor splice activation (Baralle and Baralle 2005; Buratti et al. 2007). Retention of an entire intron is less commonly reported.

The majority of disease-causing point mutations that affect splicing occur at the donor site (Krawczak et al. 2007). The results of a meta-analysis of disease-associated splicing mutations are consistent with correct donor splice site recognition being a key step in exon recognition, and that 1.6% of pathogenic missense changes in human genes are likely to affect mRNA splicing (Krawczak et al. 2007).

Splice site mutations in the *DMD* gene have been reported to result either in DMD, BMD or intermediate phenotypes in humans (Sironi et al. 2001; Adachi et al. 2003; Thi Tran et al. 2005; Takeshima et al. 2010; Magri et al. 2011), and DMD-like disease in the naturally occurring golden retriever (GRMD) canine model of DMD that arises from a single base change affecting the dystrophin exon 7 acceptor splice site (Sharp et al. 1992). The result is omission of exon 7 from the mature dystrophin transcript, and disruption of the reading frame that can be restored by exclusion of exons 6 and 8, demonstrated in vitro (McClorey et al. 2006) and in vivo (Yokota et al. 2009).

When first examining the dystrophin transcripts in cells from the DMD patients, DMD-16ss and DMD-45ss, RT-PCR across the gene lesions failed to yield reproducible amplicons, a result that is inconsistent with skipping of the respective exons. It was considered unlikely that nonsense-mediated decay alone could account for what appeared to be extremely low levels of transcript, as we routinely detect dystrophin transcripts in many different DMD patient cell and tissue samples, under equivalent amplification conditions (Arechavala-Gomeza et al. 2007; Forrest et al. 2010; Fragall et al. 2011; Adkin et al. 2012). Hence, we hypothesized that a catastrophic disruption of pre-mRNA processing may have ablated the dystrophin transcript, downstream of the gene lesion. However, amplification of the dystrophin mRNA upstream and downstream of the defects indicated that the transcript was indeed present in both cases, and the inability to generate products across the mutations was a limitation of the RT-PCR. Undertaking RT-PCR with a forward primer directed to exon 14 and reverse primers annealing within intron 16 generated amplification products of the expected size from DMD-16ss RNA, if intron 16 was retained within the mature transcript. These amplicons could not have arisen from DNA contamination as introns 14 and 15, 110 bases and about 7.6 kb, respectively, would preclude amplification under these conditions. Similar experiments, using a forward primer annealing toward the end of intron 16 and a reverse primer targeting exon 20 also generated a product of the anticipated size if intron 16 was retained within the mature mRNA.

Shorter than expected amplicons were detected in RNA extracted from untreated, and oligomer-treated DMD-16ss cells, and have been reported previously (Mitrpant et al. 2009). The smaller product detected most frequently in this study was identified by DNA sequencing, and is presumed to have arisen from endogenous skipping of exons 14, 15, and 16, as this amplicon was also detected in untreated DMD-16ss cells. Typical of the revertant fiber transcripts reported to be present in at least 50% of DMD patients (Sherratt et al. 1993; Winnard et al. 1993), this particular transcript is in-frame but occurs at a very low frequency, demonstrated by the sporadic detection at the RNA level and absence of dystrophin on a western blot.

Parallel RT-PCR studies confirmed that both ends of intron 45 were present in the dystrophin mRNA expressed in DMD-45ss cells, and primer walking indicated that at least the first and last 1 kb of intron 45 was retained. Northern blotting could confirm retention of the entire intron 45 in the mature mRNA, but was not considered necessary, as any internal cleavage should render the transcript susceptible to degradation by 5'–3'exonucleases, such as Xrn2 (Davidson et al. 2012). Furthermore, the inability to reproducibly generate amplicons across the mutations studied here is consistent with retention of a substantial portion of the introns. Consequently, the 14 kb mature dystrophin mRNA would be increased in length 2.4 and 3.5 fold, by the exon 16 and 45 splice mutations, respectively.

Transfection of the exon 16 targeting oligomer, H16A(−12 + 19), into DMD-16ss patient cells at concentrations of 10 nmol/L or above, induced transcripts missing exon and intron 16, were readily detected in a manner that did not appear dose dependent. At a transfection concentration of 5 nmol/L, the amplicon pattern was sporadic and similar to that observed in untreated cells, and thus were assumed to indicate lack of rescue of the transcript. Subsequent titration showed exon 16 skipping could be induced in DMD-16ss patient cells after transfection at lower concentrations (1–10 nmol/L), but the amplicon representing dystrophin transcripts missing exon 16 only was sporadic (data not shown). This is consistent with retention of intron 16 in the majority of the transcripts, suggesting a possible threshold effect for efficient splice switching for this mutation. Interestingly, when we first reported exon skipping in normal human myogenic cells with H16A(−12 + 19) over the oligomer transfection range 10–200 nmol/L (Harding et al. 2006), a dose response was not apparent. Subsequent titration of the oligomer in normal human myogenic cells showed a clear dose response between 0.8 and 25 nmol/L (data not shown), but this was not observed in the patient cells. The inability to reliably induce exon 16 excision in the DMD-16ss patient cells at low oligomer concentrations could reflect the influence of the donor splice site mutation on oligomer-induced modification of dystrophin splicing in this patient. This was not unexpected, as we have previously showed that small DMD mutations can influence oligomer-mediated exon skipping outcomes (Forrest et al. 2010; Fragall et al. 2011).

Mutations affecting dystrophin exon 16 are not commonly encountered and this exon would be considered to be of low priority as a therapeutic target, except to the patient and his family. Despite the rare, perhaps unique nature of the mutation reported here, this patient could be considered an excellent candidate for personalized exon skipping. Not only can the target exon be excised at high efficiency, at least in vitro, but more significantly, the loss of exon 16 does not appear to compromise dystrophin function (Schwartz et al. 2007). By chance, several members of a family were identified as missing this exon, and these asymptomatic individuals did not show elevated serum creatine kinase levels. The intent in applying exon skipping to the dystrophin transcript is to convert a DMD pre-mRNA into a BMD-like isoform, however, the loss of exon 16 from the mature dystrophin gene transcript results in a dystrophin of normal function. It is conceivable that induction of low levels of a “normal” dystrophin would confer substantial therapeutic benefit, and therefore such patients may be better candidates for clinical evaluation than those whose more common mutations only allow generation of a somewhat compromised BMD-like dystrophin isoform.

Unlike exon 16, exon 45 is within the high priority dystrophin target region for induced exon skipping. Exclusion of exon 45 is potentially applicable to an estimated 7–8% of all DMD cases (van Deutekom and van Ommen 2003; Aartsma-Rus et al. 2009). However, skipping of exon 45 alone will not be of any benefit to DMD-45ss, as dystrophin transcripts missing exon 45 are out-of-frame, and dual skipping of either exons 44 and 45 or 45 and 46 is required for reading frame rescue.

Splice-switching oligomers previously designed and optimized to excise exons 44, 45, and 46, and transfected into normal myogenic cells either individually, or in combination, induced robust, dose-dependent exon skipping. The oligomers targeting exons 44 or 46 had no detectable splice-switching effect in the DMD-45ss patient cells, and our experience with DMD-16ss suggested that intron 45 was retained in the dystrophin mRNA, precluding amplicon generation. This was subsequently confirmed by nested RT-PCR carried out from exon 43 to intron 45. The splice-switching AO H45A(−03 + 22) induced robust exon 45 skipping in normal cells at 50–100 nmol/L, with a decrease in efficiency evident at lower concentrations. When the oligomer was transfected into DMD-45ss patient cells, there was a pronounced threshold effect, with oligomer concentrations of 50 nmol/L and higher being necessary to excise exon 45 and suppress intron 45 retention in the *DMD* transcript in vitro.

Dual exon targeting efficiently excluded exons 44 and 45, and exons 45 and 46 in DMD-45ss patient cells, with readily detectable dystrophin amplicons of the expected size at transfection concentrations of 5 nmol/L (2.5 nmol/L of both oligomers) or higher. Exon 45 and 46 skipping appeared to be the more effective strategy, as determined by the relative amounts of the induced amplicon reflecting dual exon skipping. Transfection of patient cells, with either oligomer cocktail at 2.5 nmol/L did not induce any detectable product, suggesting retention of intron. Skipping of exons 44 and 45 appeared less effective, and specific RT-PCR assays confirmed the presence of some transcripts retaining intron 45. Surprisingly, the intron specific RT-PCR assays indicated some transcripts retained intron 45 despite the seemingly more efficient exon 45 and 46 skipping.

It is probable that the nature of these donor splice site mutations, together with the size of the flanking introns will impact upon oligomer-induced exon skipping. Overcoming the exon 16 gene lesion was readily achieved by skipping of this single exon, with flanking introns 15 and 16, a total expanse of 29.1 kb. In contrast, addressing the exon 45 splice mutation necessitated removal of two exons and the associated introns. Intron 44, the longest in *DMD* and perhaps all genes, is over 248 kb in length. Skipping exons 44 and 45 requires some 355 kb of pre-mRNA to be excised, whereas the alternative strategy, removing exons 45 and 46 excludes 286 kb from the primary gene transcript. Two possibilities could account for the variable dual exon skipping efficiencies. Not all oligomers excise a targeted exon with equal efficiency, exon 44 skipping in normal cells is less effective than removal of exon 46, although this is further complicated when using oligomer combinations. When assessed in normal myogenic cells, the excision of exons 44 and 45 appeared more efficient than skipping of exon 45 and 46. Another consideration is the possibility of an upper limit on the distance between exons that permit lariat formation and subsequent splicing.

In summary, we have described two *DMD* donor splice site mutations that unexpectedly led to retention of the entire downstream introns in the transcript, and some difficulty in mRNA characterization, until RT-PCR primers annealing to the introns were used. Despite this superficial similarity, the DMD-16ss gene transcript responded to single exon skipping, so that near-normal levels of dystrophin were restored in vitro*,* as assessed by western blotting. In contrast, the DMD-45ss dystrophin transcript was not amenable to single exon skipping, whereas dual skipping of exons 44 and 45 or 45 and 46 was efficiently induced, with a clear threshold effect evident. We suggest that it is likely that exon skipping strategies will need to be individualized for many of the nondeletion DMD cases.
